# Effects of Aggregate Mesostructure on Permanent Deformation of Asphalt Mixture Using Three-Dimensional Discrete Element Modeling

**DOI:** 10.3390/ma12213601

**Published:** 2019-11-02

**Authors:** Deyu Zhang, Linhao Gu, Junqing Zhu

**Affiliations:** 1 School of Transportation, Southeast University, Nanjing 210096, China; zhangdy@njit.edu.cn (D.Z.); gulinhao@seu.edu.cn (L.G.); 2 School of Architecture Engineering, Nanjing Institute of Technology, Nanjing 211167, China

**Keywords:** asphalt mixture, aggregate mesostructure, permanent deformation, discrete element method

## Abstract

This paper investigated the effects of aggregate mesostructures on permanent deformation behavior of an asphalt mixture using the three-dimensional (3D) discrete element method (DEM). A 3D discrete element (DE) model of an asphalt mixture composed of coarse aggregates, asphalt mastic, and air voids was developed. Mesomechanical models representing the interactions among the components of asphalt mixture were assigned. Based on the mesomechanical modeling, the uniaxial static load creep tests were simulated using the prepared models, and effects of aggregate angularity, orientation, surface texture, and distribution on the permanent deformation behavior of the asphalt mixtures were analyzed. It was proven that good aggregate angularity had a positive effect on the permanent deformation performance of the asphalt mixtures, especially when approximate cubic aggregates were used. Aggregate packing was more stable when the aggregate orientations tended to be horizontal, which improved the permanent deformation performance of the asphalt mixture. The influence of orientations of 4.75 mm size aggregates on the permanent deformation behavior of the asphalt mixture was significant. Use of aggregates with good surface texture benefitted the permanent deformation performance of the asphalt mixture. Additionally, the non-uniform distribution of aggregates had a negative impact on the permanent deformation performance of the asphalt mixtures, especially when aggregates were distributed non-uniformly in the vertical direction.

## 1. Introduction

With the increase of traffic and heavy vehicles, rutting has become one of the main distresses of asphalt pavement, which seriously affects driving safety and comfort. The permanent deformation behavior of asphalt mixtures has become an important issue for road researchers. Within asphalt mixtures, aggregates account for more than 90% by weight and 80% by volume. Aggregates, as the main component of asphalt mixtures, have a significant impact on the permanent deformation behavior of asphalt mixtures. Previous studies on the effects of aggregates on the permanent deformation behavior of asphalt mixtures have been mainly related to aggregate gradations. It is difficult to investigate the effects of the mesostructural characteristics of aggregates on permanent deformation behavior of asphalt mixtures, due to the limitations of available laboratory tests.

In previous studies, X-ray computed tomography (CT) techniques and image processing technology have been used for aggregate mesostructural analysis. Masad proposed computer-automated image analysis procedures to quantify the internal structure of asphalt concrete in terms of aggregate orientation, aggregate contacts, and air void distribution, and developed a finite-element model of asphalt concrete mesostructure to study the influence of localized strain distribution on the mechanical response of asphalt concrete [1,2,3]. Kose investigated strain distribution within a binder using digitized images analyzed using finite-element procedures [4]. Wang developed a method to quantify the local volume fractions of voids and their spatial gradients using X-ray tomography imaging and image analysis [5,6]. You predicted the dynamic modulus of asphalt mixtures using both the two-dimensional and three-dimensional discrete element method, generated using X-ray computed tomography [7]. However, pre-compacted specimens should have been prepared in the laboratory for control purposes in the studies mentioned above. Variability of the components within specimens have inevitably occurred, and the distribution, morphological characteristics of aggregates, and air voids are not the same even within two mixture specimens of the same kind. Therefore, there are many factors that might disrupt investigation of the effect of a single mesostructure on the behavior of asphalt mixtures. Moreover, the mesostructures of the components cannot be desirably controlled in the laboratory, and the results to date have been consequently unconvincing. Therefore, it is necessary to control the internal structures of asphalt mixtures in order to accurately analyze the effects of a single mesostructure.

At present, commonly used numerical simulation methods at the meso-scale include the finite element method (FEM) [8], the discrete element method (DEM) [9], and the combined finite–discrete element method (FDEM) [10]. In recent years, DEM, as a numerical simulation method used to solve the problem of discontinuity media, has been used to analyze the mechanical properties of asphalt mixtures. Chang developed a model called ASBAL (TRUBAL for ASphalt) based on the discrete element method by modifying the TRUBAL program to simulate hot-mix asphalt mixtures [9]. Buttlar presented a microfabric discrete-element modeling approach (MDEM) for modeling asphalt concrete mesostructures using image analysis techniques [11]. You predicted the asphalt mixture complex modulus in extension/compression across a range of test temperatures and load frequencies using the MDEM approach, and simulated and analyzed the creep responses of an asphalt mixture with a 3D-mesostructure-based DE viscoelastic model [12,13]. Abbas presented a methodology for analyzing the viscoelastic response of asphalt mixtures using the DEM [14]. Liu developed a viscoelastic model of asphalt mixtures using the discrete element method, where the viscoelastic behaviors of asphalt mastics are represented by a Burger’s model [15]. Chen built a mesomechanical model to investigate the stiffness anisotropy of asphalt concrete using the DEM [16,17]. Ma conducted simulated wheel-tracking tests on asphalt mixtures, predicted the rutting deformation of asphalt mixtures, and analyzed the mesomechanical response of aggregate skeletons within the asphalt mixtures during the tests [18,19]. Ma also investigated the effects of different parameters related to air voids on the creep behavior of asphalt mixtures based on a 3D DEM [20]. Ding proposed a new modeling method to reconstruct hollow shapes of aggregate particles using the DEM to accurately characterize the mesostructures of aggregates and efficiently predict the mechanical properties of aggregate skeletons [21]. It has been proven that the DEM can accurately capture the internal structure of asphalt mixtures, and desirably control the mesostructural characteristics of aggregates, asphalt binder, and air voids within the mixtures [22,23,24,25]. However, few researchers have focused on the permanent deformation behavior of asphalt mixtures, especially the effects of aggregate mesostructure on the permanent deformation behavior of asphalt mixtures using the DEM.

The objective of this study was to investigate the effects of aggregate mesostructures on the permanent deformation behavior of asphalt mixtures using the DEM. A 3D DE model of asphalt mixture including aggregates, asphalt mastics, and air voids was developed using the DEM software Particle Flow Code in three dimensions (PFC3D). Three-dimensional discrete element simulations of uniaxial static creep tests of asphalt mixtures were carried out. By accurately controlling the mesostructural characteristics of the aggregates, such as angularity, orientation, surface texture, and distribution, the effects of aggregate mesostructures on the permanent deformation behavior of the asphalt mixtures were carefully analyzed.

## 2. Materials and Methods

### 2.1. Materials

**Figure 1:**
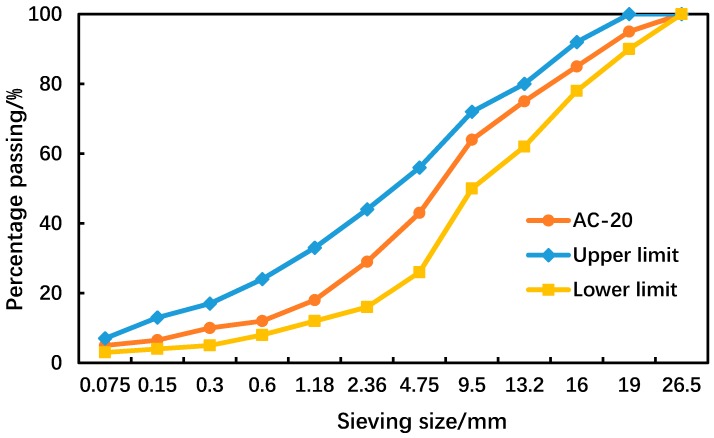
Gradation of the AC-20 asphalt mixture.

According to the Marshall mix design method based on the Chinese specifications [26], a dense-graded asphalt mixture, AC-20, was prepared in the laboratory. Aggregate gradation with a nominal maximum aggregate size of 19 mm is shown in Figure 1. Based on the Marshall mix design method, the volumetric properties of the AC-20 asphalt mixture were determined as shown in Table 1. Aggregates smaller than 2.36 mm, mineral filler, and asphalt binder were mixed as asphalt mastic with an asphalt content of 11.5%. The air void content of the asphalt mastic was supposed to be zero to maximize its flowability.

### 2.2. Laboratory Tests

**Figure 2:**
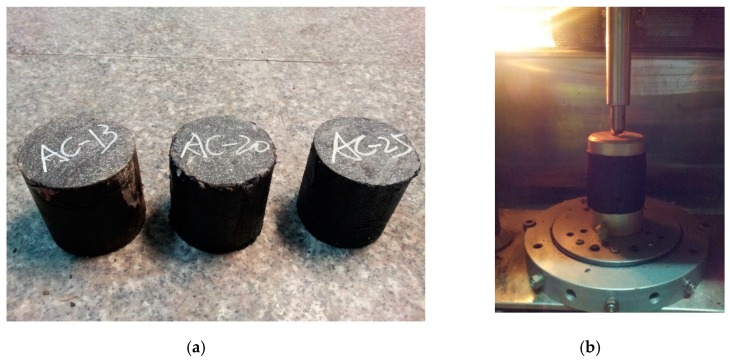
(**a**) The asphalt mastic specimens; (**b**) the uniaxial static creep test testing process.

The uniaxial static creep test is one of the simplest and most practical test methods, and it is an effective way to investigate the permanent deformation behaviors of asphalt mixtures and mastics at high temperatures. In this test, an instantaneous load is applied to a cylinder specimen in the axial direction and the load is kept constant, and the creep curve of the asphalt mixtures or mastics can be obtained. Uniaxial static creep tests were conducted in this paper to evaluate the permanent deformation behaviors of asphalt mixtures and mastics at the temperature of 60 °C using a universal test machine (UTM, IPC Global, Melbourne, Australia) [27]. Cylindrical specimens of asphalt mixtures with diameter of 100 mm and height of 150 mm were prepared using a gyratory compactor for the uniaxial static creep test. Cylindrical asphalt mastic specimens with diameter and height of 100 mm were prepared by vibration. The applied axial stresses for the asphalt mixture and asphalt mastic were set at 0.7 MPa and 0.07 MPa, respectively, in this study. The asphalt mastic specimens and the testing process are shown in Figure 2. Lab test results were used for parameters of DE modeling and to be compared with simulation test results, and are presented in the following sections. The experimental program for the asphalt mixture and mastics is summarized in Table 2.

## 3. Discrete Element Modeling of Asphalt Mixtures

### 3.1. Discrete Element Modeling

An asphalt mixture, as a multiphase composite, is composed of aggregates, asphalt binder, and air voids. If the particle size of the fine aggregates and mineral filler is small, it will lead to a large increase of discrete elements in the DE model, which is bound to significantly reduce the computational efficiency if the fine aggregate and mineral powder are considered fully in DE models of asphalt mixtures. Therefore, the asphalt mixture was simplified into coarse aggregates (bigger than 2.36 mm), asphalt mastics (asphalt binder, fine aggregates smaller than 2.36 mm and mineral filler), and air voids to improve the calculation efficiency. The asphalt mastic was considered as a homogeneous material within the DE model.

**Figure 3:**
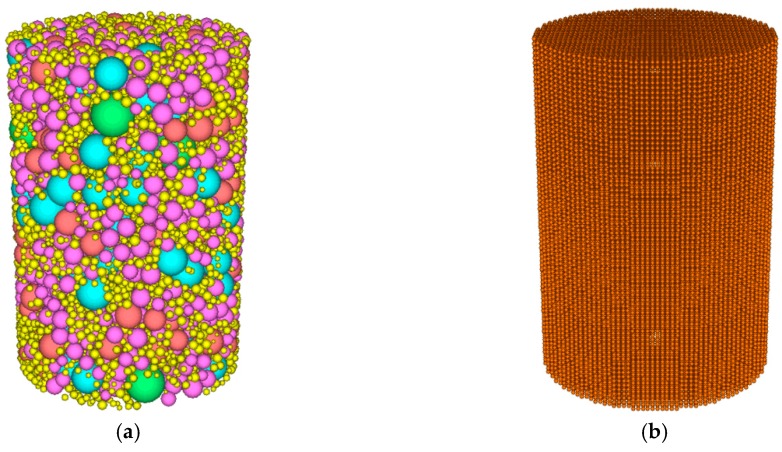
(**a**) Graded coarse aggregates; (**b**) uniform-sized packed discrete elements.

During modeling, the spatial range of the mixture model was first constructed by “wall”. The quantity of coarse aggregates in each grade was calculated according to aggregate gradation, asphalt content and air void content. The coarse aggregate balls were then delivered into the spatial range of the mixture model constructed by “wall”. Force was generated between overlapped balls, as there were overlaps between the coarse aggregate balls during delivery. This force was eliminated by the “cycle” command. The coarse aggregate balls within the spatial range of the mixture model are shown in Figure 3a.

A regular array of discrete elements was then filled into the mixture space as the base of coarse aggregates and asphalt mastic, as shown in Figure 3b. The radius of the discrete element was set to 1 mm, considering the calculation efficiency.

**Figure 4:**
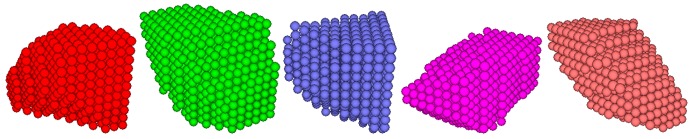
Irregularly shaped polyhedron aggregates.

**Figure 5:**
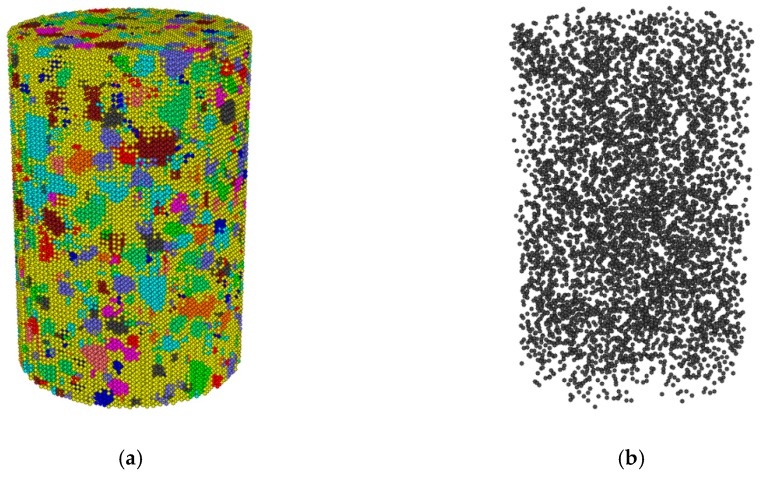
(**a**) Discrete element (DE) model of asphalt mixture; (**b**) air voids within the DE model.

It has been proven that the geometry of coarse aggregates has a significant effect on the permanent deformation behavior of asphalt mixtures [28,29]. Therefore, the coarse aggregate geometry will directly determine the accuracy of the simulation results. In this paper, coarse aggregate was simplified as an irregular polyhedron. Random planes were used to cut a cube or a sphere to generate irregular polyhedral aggregates using a user-defined program. By traversing the regularly packed discrete elements, the positional relationship between the regular packing discrete elements and the irregular polyhedral aggregates could be evaluated. Discrete elements belonging to irregular polyhedral aggregate were regarded as aggregate elements, and were set as a clump, as seen in Figure 4. The original coarse aggregate balls were then deleted. Discrete elements outside the irregular polyhedron aggregate were considered as asphalt mastic. The initially developed 3D DE model of asphalt mixture is shown in Figure 5a.

Considering the complexity of air void distribution within an asphalt mixture specimen, the air voids were assumed to be randomly distributed [30]. Asphalt mastic elements in the model were traversed and randomly deleted and regarded as air voids. The distribution of air voids is shown in Figure 5b.

### 3.2. Mesomechanical Models and Parameters

**Figure 6:**
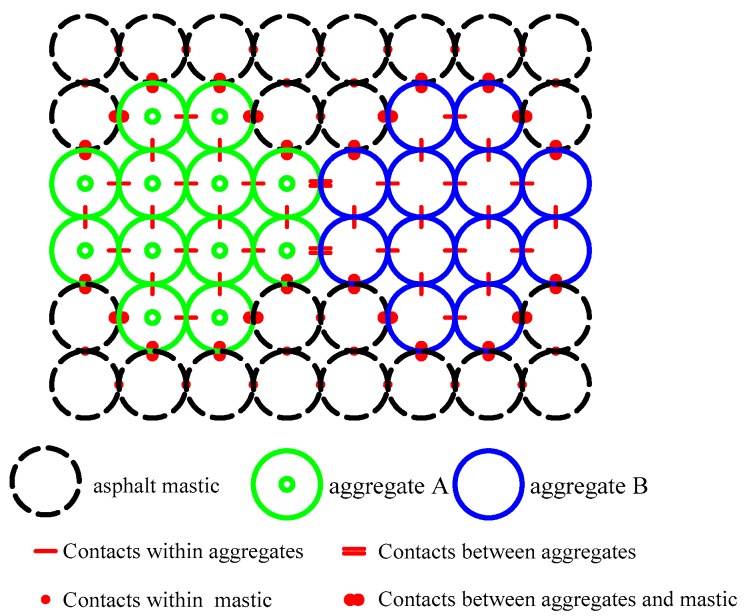
Interactions among the components within the asphalt mixture.

There are four types of contact within asphalt mixtures, including contacts between aggregate elements, contacts between adjacent aggregates, contacts between asphalt mastic elements, and contact between asphalt mastic and aggregates, as seen in Figure 6. In PFC3D, mesomechanical models are used to describe the contact behaviors between different components within asphalt mixtures. In this study, the mesomechanical models used in the model of asphalt mixtures included the stiffness model, slipping model, bonding model, and Burger’s model.

Due to the high stiffness of an aggregate, it can be approximately regarded as an elastic material. In this study, the stiffness model and slipping model were used to characterize the mesomechanical behavior between adjacent aggregates. As coarse aggregates within the model of asphalt mixtures were set as clumps, it was unnecessary to assign the mesomechanical model within aggregates. The mesomechanical parameters of the stiffness model could be obtained from the macro properties of the aggregates, as shown in Equations (1) and (2) [15,31,32]. The macro parameters for the aggregates are shown in Table 3 [9,12,33].

(1) $$E=\frac{k_{n}}{4R},\;k_{s}=\frac{k_{n}}{2\left(1+\upsilon \prime \right)}$$

(2) $$\mu_{c}=\mu_{a}$$

where *E* is the apparent Young’s modulus of the aggregates, *k_n_* and *k_s_* are the stiffness in the normal and shear direction, respectively, *R* is the discrete element radius, *υ*′ is the aggregate Poisson’s ratio, *μ_c_* is the friction coefficient between aggregates, and *μ_a_* is the friction coefficient of aggregates.

**Figure 7:**
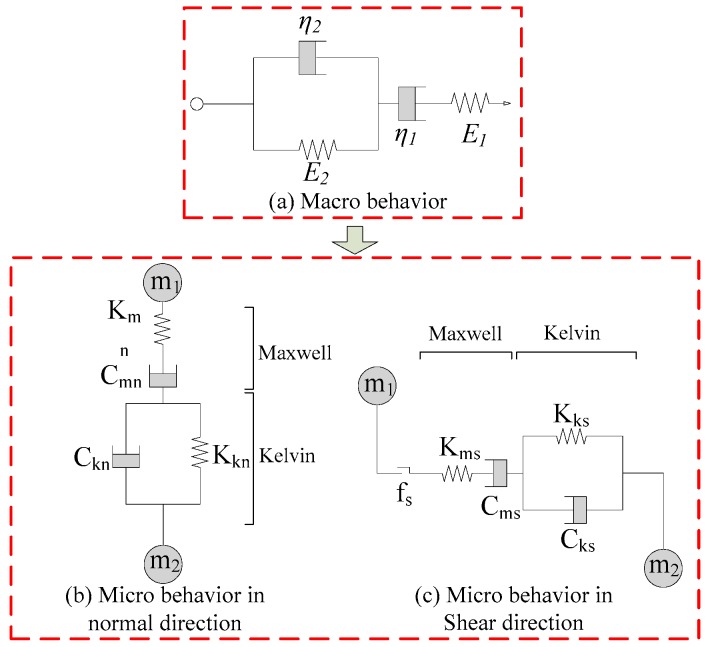
Burger’s models. (**a**) Macro behavior; (**b**) micro behavior in the normal direction; (**c**) micro behavior in the shear direction.

Asphalt mixtures show a macro viscoelastic behavior due to the viscoelastic characteristic of the asphalt mastic. Therefore, the mesomechanical model of the asphalt mastic directly affects the macro properties of asphalt mixtures. The meso Burger’s model in PFC3D was well able to describe the mechanical properties of viscoelastic materials and was used to characterize the viscoelastic properties of the asphalt mastic in this study, as shown in Figure 7. It has been proven that there is a conversion between the parameters of the meso Burger’s model and the macro Burger’s model [15,31], as shown in Equations (3) and (4). Parameters of meso Burger’s model could then be obtained from the macro Burger’s model parameters. The macro Burger’s model parameters of asphalt mastic were obtained by uniaxial static creep test, as shown in Table 3.

(3) $$K_{mn}=E_{1}L,\;C_{mn}=\eta_{1}L,\;K_{kn}=E_{2}L,\;C_{kn}=\eta_{2}L$$

(4) $$K_{ms}=\frac{E_{1}L}{2\left(1+\upsilon \right)},\;C_{ms}=\frac{\eta_{1}L}{2\left(1+\upsilon \right)},\;K_{ks}=\frac{E_{2}L}{2\left(1+\upsilon \right)},\;C_{ks}=\frac{\eta_{2}L}{2\left(1+\upsilon \right)}$$

where *E*_1_, *η*_1_, *E*_2_, and *η*_2_, are parameters of the macro Burger’s model; *K_mn_*, *C_mn_*, *K_kn_*, and *C_kn_* are parameters of the meso Burger’s model in the normal direction; *K_ms_*, *C_ms_*, *K_ks_*, and *C_ks_* are parameters of the meso Burger’s model in the shear direction; *L* is the distance between adjacent discrete elements; and *υ* is Poisson’s ratio of the asphalt mastic.

**Figure 8:**
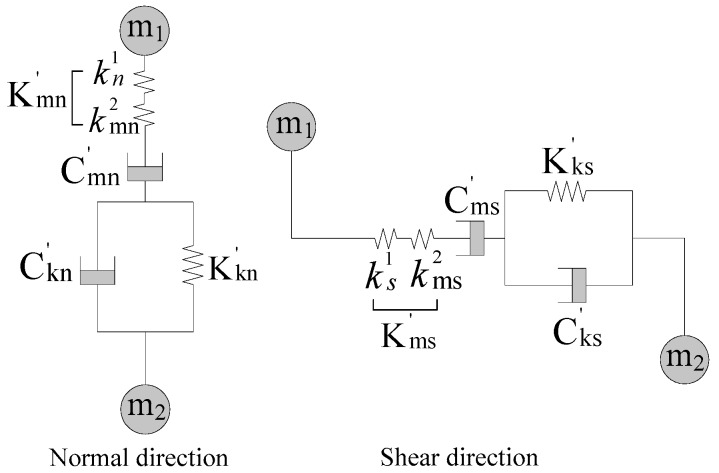
Equivalent meso Burger’s model for contacts between aggregates and asphalt mastic.

The contact behavior between aggregates and the asphalt mastic can be characterized by the equivalent meso Burger’s model, as shown in Figure 8. The equivalent meso model parameters can be obtained from the macro parameters of the aggregates and asphalt mastic, as expressed in Equations (5) and (6) [15,31]:

(5) $${K^{\prime }}_{mn}=\frac{2EE_{1}}{E+E_{1}}L,\;{C^{\prime }}_{mn}=2\eta_{1}L,\;{K^{\prime }}_{kn}=2E_{2}L,\;{C^{\prime }}_{kn}=2\eta_{2}L$$

(6) $${K^{\prime }}_{ms}=\frac{2EE_{1}}{E\left(1+\upsilon \right)+2E_{1}\left(1+\upsilon \right)}L,\;{C^{\prime }}_{ms}=\frac{\eta_{1}L}{\left(1+\upsilon \right)},\;{K^{\prime }}_{ks}=\frac{E_{2}L}{\left(1+\upsilon \right)},\;{C^{\prime }}_{ks}=\frac{\eta_{2}L}{\left(1+\upsilon \right)}$$

where *K*′*_mm_*, *C*′*_mm_*, *K*′*_kn_*, and *C*′*_kn_* are the parameters of the equivalent meso Burger’s model between the aggregates and mastic in the normal direction and *K*′*_ms_*, *C*′*_ms_*, *K*′*_ks_*, and *C*′*_ks_* are the parameters of the equivalent meso Burger’s model between the aggregates and the mastic in the shear direction.

### 3.3. Simulation of Uniaxial Creep Test

**Figure 9:**
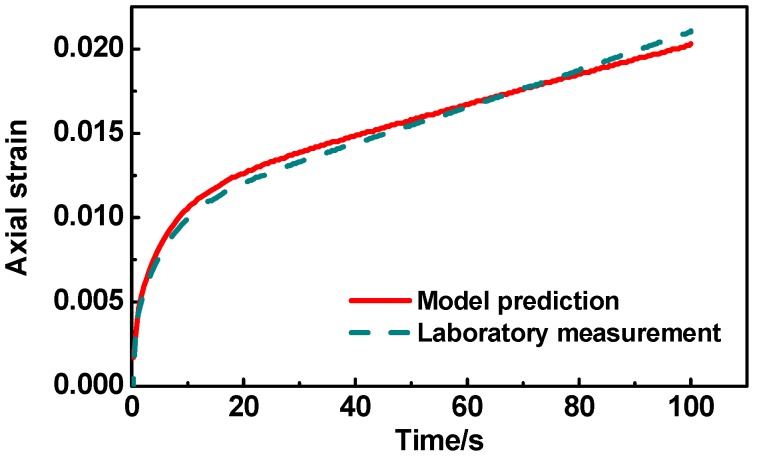
Results of discrete element model (DEM) prediction and laboratory measurement.

Based on the above-mentioned DE modeling procedures for asphalt mixtures, the simulated uniaxial creep test was carried out using PFC3D. The results were compared with laboratory test results under the same conditions. Figure 9 shows the axial strain curve obtained from the simulation and lab test, and it can be seen that the simulation curve was very close to the curve of the laboratory test. This indicates that the simulation of uniaxial creep test conducted using DEM could precisely estimate the permanent deformation behavior of asphalt mixtures.

## 4. Results and Discussion

### 4.1. Effect of Aggregate Angularity

The aggregate angularity could represent the morphological characteristics of aggregates, and significantly affect the skeleton stability, interlocking force, and shear resistance of aggregates. It has been proven that the aggregate angularity is closely related to the strength of asphalt mixtures, especially the permanent deformation resistance and shear resistance [28,29].

Some previous studies about aggregate angularity have been carried out, and some indexes to describe the aggregate angularity have been proposed. These angularity indexes are mainly divided into two categories, one denoting the roundness of aggregate corners and the other one denoting the roundness of the overall outline of the aggregate [34]. However, most of these angularity indexes are in two dimensions. Moreover, the measurement and calculation of the first type of angularity index is very complex, while the second type could be affected by the overall outline of aggregates. Therefore, the angularity indexes mentioned above cannot fully represent the angularity characteristics of aggregates. In this study, the ratio of surface area of irregular aggregate to equivalent ellipsoid was proposed to characterize the aggregate angularity. The equivalent ellipsoid of an aggregate has the same volume as the aggregate. It was considered that the equivalent ellipsoid of aggregates could accurately reflect the overall outline, and this angularity index could significantly reduce the influence of aggregate outline on angularity index, as expressed in Equation (7). The larger *AI* is, the better the aggregate angularity is.

(7) $$AI={\left(\frac{S}{Sellipse}\right)}^{3}$$

where *AI* is the angularity index of aggregates, *S* is surface area of aggregates, and *S_ellipse_* is surface area of equivalent ellipsoid.

As mentioned above, irregular polyhedral aggregates were generated by cutting a cube or a sphere with random planes. Thus, the equivalent ellipsoid of an irregular polyhedral aggregate can be approximated to an equivalent sphere, and then Equation (8) can be further expressed as follows:

(8) $$AI={\left(\frac{S}{Ssphere}\right)}^{3}={\left(\frac{S}{4\pi {\left(\sqrt[{3}]{3V/4\pi }\right)}^{2}}\right)}^{3}=\frac{S^{3}}{36\pi V^{2}}$$

where *S_sphere_* is surface area of equivalent sphere and *V* is the volume of aggregates.

**Figure 10:**
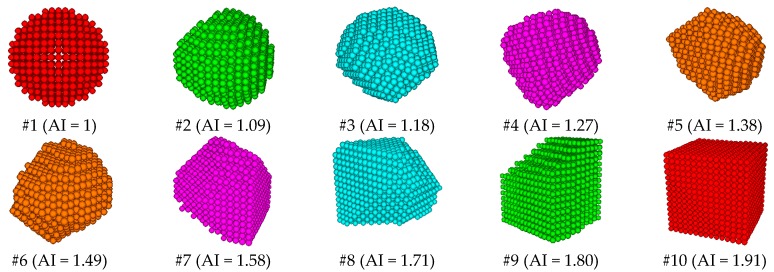
Irregular polyhedral aggregates with different angularities. AI: angularity index.

Coarse aggregates with different angularities were generated by cutting a cube or a sphere with random planes. Some coarse aggregates with different angularities are shown in Figure 10, in which their angularity become better with the increasing number. DE models of asphalt mixtures with different aggregate angularities were then obtained.

**Figure 11:**
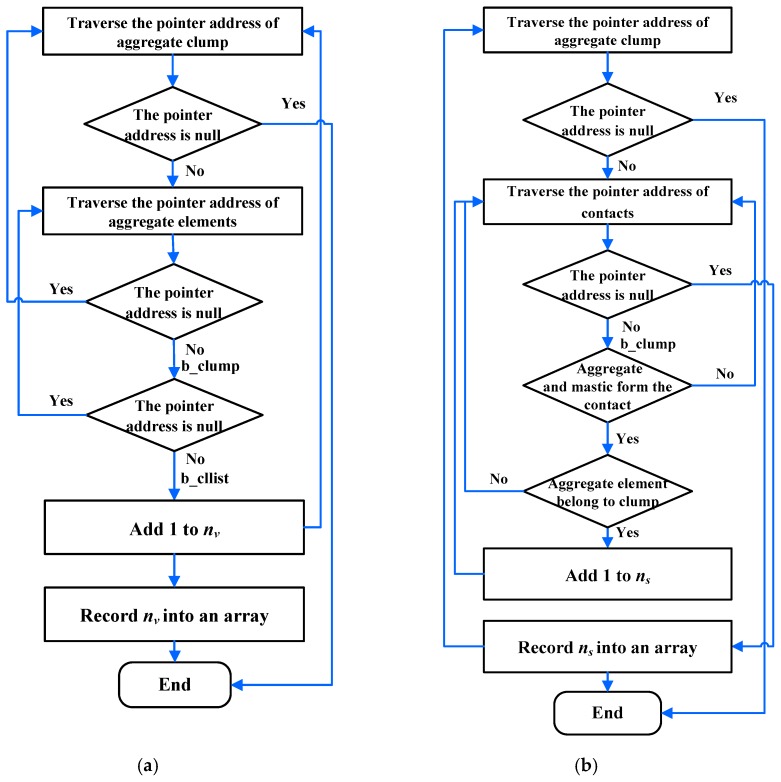
Procedure for quantifying the discrete element number of aggregates: (**a**) quantifying the number of aggregate surfaces; (**b**) quantifying the number that makes up aggregates.

In order to calculate the surface area of aggregates, the number of discrete elements on aggregate surface and the number of discrete elements that made up the aggregates were counted. The angularity index of the aggregates was then calculated by Equation (9). The method used to count the number of discrete elements on the aggregate surface and the number of discrete elements that make up aggregates is shown in Figure 11.

(9) $$AI=\frac{{\left(4R^{2}n_{S}\right)}^{3}}{36\pi {\left(8R^{3}n_{V}\right)}^{2}}=\frac{{n_{S}}^{3}}{36\pi {n_{V}}^{2}}$$

where *n_s_* is the number of discrete elements on the aggregate surface, *n_v_* is the number of discrete elements that make up the aggregate, and *R* is radius of the discrete element.

The angularity of a single aggregate can be obtained by Equation (9). However, there are many aggregates within asphalt mixtures, and the angularities of each aggregate are different. It is necessary to evaluate the overall angularity of the aggregates within asphalt mixtures. In this study, the volumetrically weighted average of the angularities of each aggregate was used to describe the overall angularity of the aggregates within the asphalt mixtures, as expressed in Equation (10).

(10) $$\bar{AI}=\frac{\sum_{i=1}^{n}Vi\times AIi}{\sum_{i=1}^{n}Vi}$$

where $\bar{AI}$ is the overall angularity of aggregates, *AI_i_* is the angularity of aggregate *i*, *V_i_* is the volume of aggregate *i*, and *n* is the number of aggregates.

**Figure 12:**
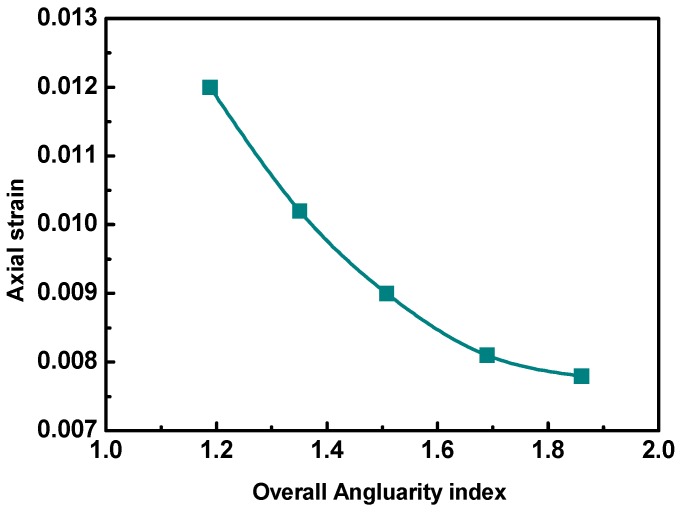
Axial strains with different overall aggregate angularities.

Simulations of uniaxial static creep tests were then conducted on the DE models of asphalt mixtures with different overall aggregate angularities. The results are shown in Figure 12. The permanent deformation performance of asphalt mixtures improved with the increasing aggregate angularity, but the improvement weakened gradually. The axial deformation of the asphalt mixture was the smallest when the aggregates were closest to a cube. This is consistent with the requirement of selecting aggregates with shapes close to a cube. Therefore, aggregates with good angularities should be selected to improve the permanent deformation resistance of asphalt pavement.

### 4.2. Effect of Aggregate Orientation

**Figure 13:**
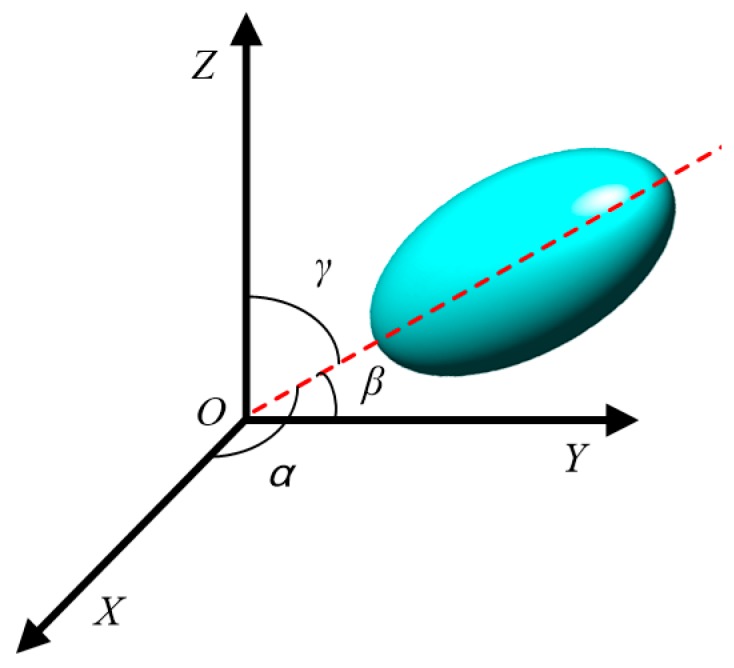
Orientation of ellipsoidal aggregate.

Previous studies about aggregate orientation have generally defined the long axis of the equivalent ellipse of aggregate in two dimensions as the long axis of aggregate, and used the angle between the long axis and the horizontal direction to represent the aggregate orientation [18,19,20,21]. In this study, the aggregate orientation was defined in three dimensions. The long axis of the equivalent ellipsoid of the aggregate was taken as the long axis, and the angles *α*, *β*, and *γ* between the long axis and X axis, Y axis, and Z axis were used to represent the aggregate orientation in three dimensions, as shown in Figure 13.

**Figure 14:**
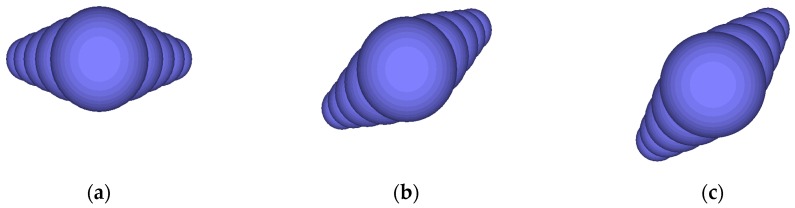
Ellipsoidal aggregates with different orientations: (**a**) 0°; (**b**) 30°; (**c**) 45°.

In order to investigate the effect of aggregate orientation on the permanent deformation performance of asphalt mixtures, a subroutine was written in PFC3D with the built-in “Fish” language. The long axis of the aggregate was set to be parallel to the YOZ surface, and the aggregate orientation was described by the angle between the long axis of the aggregate and the XOY surface. Some coarse aggregates with different orientations are shown in Figure 14.

Graded coarse aggregates with different orientations were generated and randomly put into the cylinder space of the asphalt mixture model. The unbalanced forces between the aggregates were eliminated by the “cycle” command, so that the coarse aggregates within the asphalt mixtures could reach equilibrium. The angular velocities of the coarse aggregates were fixed to keep the aggregate orientations constant during modeling.

**Figure 15:**
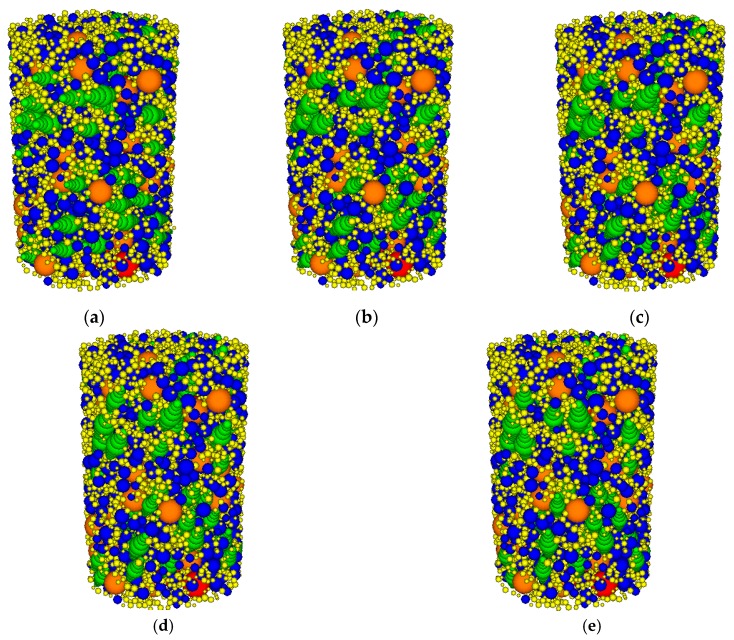
Coarse aggregates with different aggregate orientations within asphalt mixtures: (**a**) 0°; (**b**) 30°; (**c**) 45°; (**d**) 60°; (**e**) 90°.

The effects of the orientations of aggregates of different sizes on the mechanical properties of the asphalt mixtures are different. To address the size effect, 30 cylinder DE models of asphalt mixtures with different orientations of aggregates with different sizes were developed. The aggregate orientations were varied from 0°, 30°, 45°, 60°, to 90°. The rest of the aggregates remained spherical. Figure 15 shows the coarse aggregates with different orientations.

**Figure 16:**
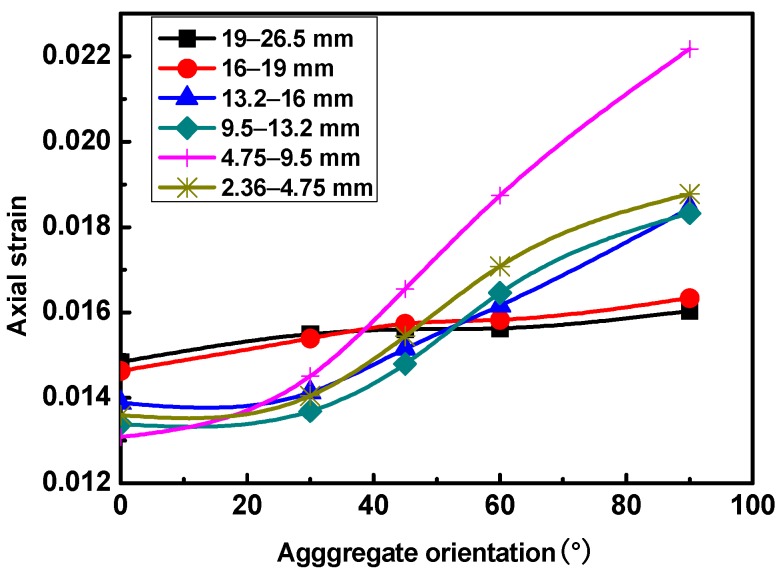
Axial strains with different aggregate orientations.

Simulations of uniaxial static creep tests were then conducted on the prepared DE models. The results are shown in Figure 16.

It can be seen from Figure 16 that the axial strains of the mixtures increased gradually with the increasing aggregate orientations. The orientations of 19–26.5mm and 16–19 mm aggregates had insignificant effects on the permanent deformation behavior of asphalt mixtures. The orientations of 13.2–16 mm, 9.5–13.2 mm, and 2.36–4.75 mm aggregates had more significant effects than those of 19–26.5 mm and 16–19 mm. The orientation of 4.75–9.5 mm aggregates had the greatest effect on the permanent deformation behavior of the asphalt mixtures. The reason is that the aggregates tended to rotate horizontally under vertical loads when the aggregate orientation was large. Thus, the aggregate skeleton was unstable and led to large axial deformation of the asphalt mixtures. When the aggregate orientation was closer to horizontal, the aggregate skeleton was more stable, and the axial deformation of the mixture specimens was smaller. It has been shown that the percentage pass of 4.75 mm plays an important role in controlling the aggregate skeleton, and the influence is next to that of air void content on the permanent deformation behavior of asphalt mixtures. This may be the reason why the orientation of 4.75–9.5 mm aggregates had such a great influence. Therefore, different kinds of rollers should be used during rolling compaction of asphalt pavement, which could force the aggregate orientation to be more horizontal. Thus, the asphalt pavement would reach a more stable state and its permanent deformation performance could be significantly improved.

### 4.3. Effect of Aggregate Surface Texture

**Figure 17:**
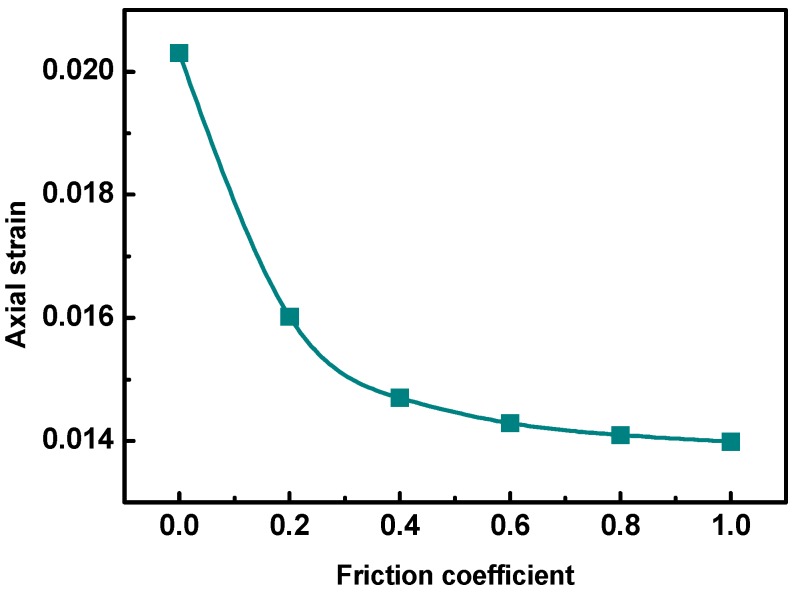
Axial strains with different aggregate surface textures.

The surface texture of aggregates reflects the roughness of aggregate surface. The rougher the aggregate surface, the better the surface texture. Internal friction between aggregates and adhesion between aggregate and asphalt binder are closely related to the surface texture of aggregates. It has been proven that the surface texture of aggregates has a significant impact on the performance of asphalt mixtures, especially the permanent deformation performance. The randomly created irregular polyhedral aggregates in the DE model could only reflect geometry and angularity, without characterizing the surface texture of aggregates. In this study, the friction coefficient between aggregates was used to indirectly represent the surface texture of aggregates. The higher the friction coefficient, the rougher the surface texture. A series of DE models of asphalt mixture specimens with different aggregate friction coefficients assigned were developed. The internal structures of the developed DE models were the same, and only the aggregate friction coefficients of polyhedron aggregates in the DE models were different. Simulations of uniaxial static creep tests were conducted on the prepared DE models. The results are shown in Figure 17.

It can be seen from Figure 17 that the permanent deformation performance of the asphalt mixtures was improved with the increasing friction coefficient, but the increment was gradually lowered. The permanent deformation resistance of the asphalt mixtures increased with rougher aggregate surface texture. Therefore, aggregates with good surface texture should be used to improve the permanent deformation performance of asphalt pavement.

### 4.4. Effect of Aggregate Distribution

Aggregates are ideally uniformly distributed within asphalt mixture for optimum performance. However, insufficient mixing force or mixing time may cause non-uniform distribution of aggregates. Aggregate segregation may also occur during paving. Early distresses of asphalt pavement such as cracking, pothole, blooding, and rutting are closely related to the material inhomogeneity caused by variable construction quality. The non-uniform distribution of aggregates greatly influences the internal structure of asphalt mixtures and consequently lowers the permanent deformation performance of asphalt mixtures.

**Figure 18:**
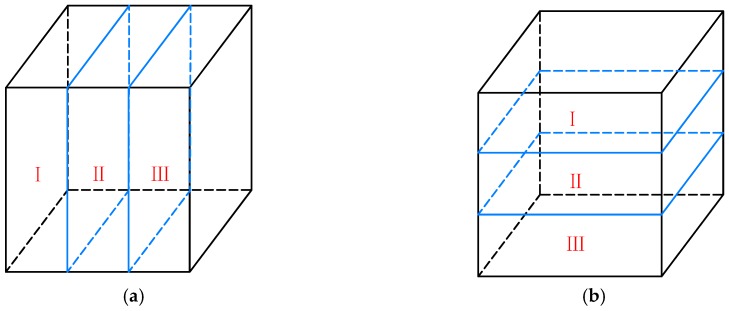
Partitioning within asphalt mixture specimen: (**a**) partitioning in the horizontal direction; (**b**) partitioning in the vertical direction.

**Figure 19:**
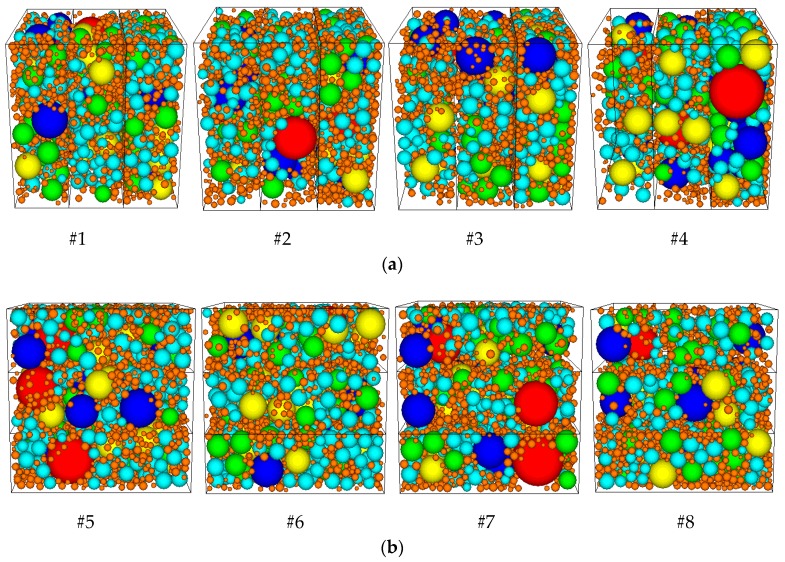
Aggregates distributed non-uniformly within DE models of asphalt mixtures: (**a**) aggregates distributed non-uniformly in the horizontal direction; (**b**) aggregates distributed non-uniformly in the vertical direction.

To evaluate the uniformity of aggregate distribution, a cubic space containing asphalt mixtures was constructed by “wall” and the specimen was divided into three parts evenly in both the horizontal and vertical directions, as shown in Figure 18. Coarse aggregates with different sizes were added to each of the three parts, as shown in Figure 19 to create non-uniform distribution. The total volume of aggregates within the three parts remained constant. The distribution percentages of the aggregate volume within each part are shown in Table 4.

A quantitative indicator of aggregate distribution was proposed to measure the uniformity of distribution of aggregate. The volume of aggregates within each part was recorded and the coefficient of variation (*var*) of aggregate distribution was calculated as follows:

(11) $$Vj=\overset{m}{\underset{i=1}{\sum }}Vi$$

(12) $$\bar{V}=\overset{n}{\underset{j=1}{\sum }}Vj/n$$

(13) $${var}_{j}=\sqrt{{\left(Vj-\bar{V}\right)}^{2}/\left(n-1\right)}/\bar{V}$$

(14) $$var=\overset{n}{\underset{j=1}{\sum }}varj$$

where *V_i_* is the volume of aggregate *i* within part *j*, *V_j_* is the total volume of aggregates within part *j*, $\bar{V}$ is the average aggregate volume of all divided parts, *var_j_* is the coefficient of variation of the total volume of aggregates within part *j*, and *var* is the coefficient of variation of the total volume of aggregates within specimen.

**Figure 20:**
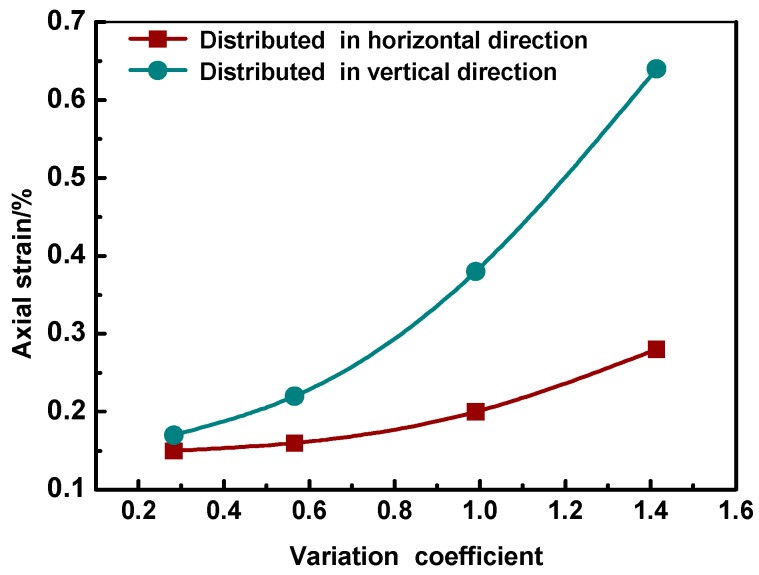
Axial strains with different coefficient of variation of aggregate distribution.

Figure 20 and Table 5 below present the results, in which the axial strain was calculated by measuring the axial deformation and dividing it by the initial axial length.

As shown in Figure 20 and Table 5, axial deformation of the specimens increased as the coefficient of variation of aggregate distribution increased using both partition methods. Aggregate distribution had a significant impact on the deformation performance of the asphalt mixtures. Non-uniformity in the vertical direction had much more impact on the deformation of the specimen than that in the horizontal direction, as shown in Figure 20.

## 5. Conclusions

In this study, three-dimensional discrete element (DE) models of asphalt mixtures composed of coarse aggregates, asphalt mastic, and air voids was developed using PFC3D, and uniaxial static load creep tests were conducted on the prepared models to investigate the effects of aggregate mesostructure on the permanent deformation behavior of the asphalt mixtures. The effects of aggregate angularity, orientation, surface texture, and distribution on the permanent deformation behavior of asphalt mixtures were carefully analyzed. The following conclusions were drawn:

(1) The constructed DE models of asphalt mixtures could accurately capture the mesostructures of aggregates, such as angularity, orientation, surface texture, and distribution. The simulation of the uniaxial creep test conducted using the DEM could precisely estimate the permanent deformation behavior of asphalt mixtures.

(2) The permanent deformation performance of the asphalt mixtures was improved with increasing aggregate angularity. The axial deformation of the asphalt mixtures was smallest when the aggregates were close to cubical. Good aggregate angularity had a positive effect on the permanent deformation performances of asphalt mixtures, especially when the aggregates were nearly cubical.

(3) Aggregate packing was more stable when the orientations tended to be horizontal, which improved the permanent deformation performances of asphalt mixtures. The percentage pass of 4.75 mm played an important role in controlling the aggregate skeleton and had a great impact on the permanent deformation behaviors of the asphalt mixtures.

(4) The permanent deformation performance of the asphalt mixtures was improved with increasing friction coefficient, indicating that aggregates with good surface texture benefitted the permanent deformation performance of the asphalt mixtures.

(5) Axial deformation of the asphalt mixture specimen increased with increasing coefficient of variation of aggregate distribution. Non-uniform distribution of the aggregates had a negative impact on the permanent deformation performance of the asphalt mixtures, especially when the aggregates were distributed non-uniformly in the vertical direction.

In the future research steps, a viscoplastic mesomechanical model will be developed to further describe the viscoplastic behavior of the permanent deformation behavior of asphalt mixtures.

## Figures and Tables

**Table 1:** Volumetric properties of the AC-20 asphalt mixture.

Asphalt Content (%)	Bulk Density (g/cm^3^)	VV (%)	VMA (%)	VFA (%)
4.3	2.432	4.0	13.8	72.2

**Table 2:** Experimental program for the asphalt mixture and mastics.

Materials	Specimen Size	Test	**Load/MPa**
Asphalt mixture	Φ 100 mm × H 150 mm (cylinder) L 100 mm × L 150 mm (cubic)	Uniaxial static creep test	0.7
Asphalt mastic	Φ 100 mm × H 100 mm (cylinder)	Uniaxial static creep test	0.07

**Table 3:** Macro parameters for asphalt mastic and aggregates.

*E*_1_ (MPa)	*η*_1_ (MPa·s)	*E*_2_ (MPa)	*η*_2_ (MPa·s)	*υ*	*E* (GPa)	*μ_a_*	*υ*′
0.568	973.163	0.396	27.895	0.5	55.5	0.5	0.35

**Table 4:** Volume distribution percentages within different parts.

Model No.	Volume Distribution Percentage within Each Part (%)		
	Part I	Part II	Part III
Partitioned in the horizontal direction			
#1	30	30	40
#2	25	30	45
#3	20	30	50
#4	15	30	55
Partitioned in the vertical direction			
#5	30	30	40
#6	25	30	45
#7	20	30	50
#8	15	30	55

**Table 5:** Axial strains when aggregates were distributed non-uniformly.

Model No.	Coefficient of Variation	Axial Strain ε (%)	ε/ε_0_
Partitioned in the horizontal direction			
#1	0.283	0.15	1.12
#2	0.495	0.16	1.24
#3	0.707	0.20	1.56
#4	0.919	0.28	2.15
Partitioned in the vertical direction			
#5	0.283	0.17	1.31
#6	0.495	0.22	1.70
#7	0.707	0.38	2.90
#8	0.919	0.64	4.90
